# Fear of COVID-19, Perceived Stress, and PTSD: The Serial Mediating Role of Sense of Coherence

**DOI:** 10.3390/ejihpe13110169

**Published:** 2023-10-31

**Authors:** Anita Padmanabhanunni, Tyrone Brian Pretorius

**Affiliations:** Department of Psychology, University of the Western Cape, Bellville 7530, South Africa; apadmana@uwc.ac.za

**Keywords:** serial mediation, fear of COVID-19, perceived stress, sense of coherence, post-traumatic stress disorder

## Abstract

The literature has identified that a sense of coherence plays a protective role in the relationship between adverse events and mental health. The current study examines the role of a sense of coherence (SOC) in the relationship between fear of COVID-19, perceived stress, and dimensions of post-traumatic stress disorder (PTSD). Participants (n = 322) were students at a metropolitan university in South Africa who completed the Fear of COVID-19 Scale, the Perceived Stress Scale, the 13-item Sense of Coherence Scale, and the PTSD Checklist. Path analysis was used to conduct a serial mediation analysis. The results show that SOC mediates the relationship between perceived stress and the dimensions of PTSD but does not mediate the relationship between fear of COVID-19 and PTSD. Furthermore, the relationship between fear of COVID-19 and dimensions of PTSD was mediated by serial perceived stress and sense of coherence, supporting the hypothesis that higher levels of fear of COVID-19 leads to higher levels of perceived stress. However, while high levels of fear of COVID-19 increase perceived stress, SOC significantly mediates the subsequent impact on PTSD symptoms.

## 1. Introduction

The COVID-19 pandemic, caused by the novel coronavirus SARS-CoV-2, has had an unparalleled impact on global health systems, economies, and daily life. Despite widespread awareness of the disease and general support for public health measures to contain it, the implementation of social distancing and lockdown protocols, coupled with the potentially severe outcomes associated with infection, contributed to elevated levels of stress and adverse mental health outcomes among many population groups [1,2,3]. Fear remained the most common response to the pandemic and this was manifested as heightened anxiety, social withdrawal, and compliance with or resistance to public health measures [4]. The pervasiveness of this fear led not only to immediate behavioral changes—such as the increased use of masks, hand sanitizers, and social distancing—but also to significant shifts in social dynamics, such as heightened stigmatization of infected individuals or those perceived as high-risk individuals [5]. The ever-present fear of contracting the virus or transmitting it to others, along with changes in work–life dynamics, quarantine, and ongoing adjustments to governmental policies, made the fear of COVID-19 a significant catalyst for increased stress perception among vast sections of the global population [6,7]. Perceived stress refers to the subjective evaluation of the nature and severity of stressors [8].

The persistent fear associated with the outbreak of the disease has been found to lead to symptoms commonly associated with PTSD, such as intrusive thoughts, heightened anxiety, and emotional numbing. A meta-analysis focusing on PTSD among schoolteachers [9] reported an 11% prevalence rate for the disorder. This was attributed to the re-opening of schools during the outbreak and the increased risk of contagion among teachers, which aggravated fear and anxiety and contributed to PTSD symptomology. Ochnik and colleagues [10] investigated longitudinal predictors of COVID-19-related PTSD among university students in Poland, Germany, Slovenia, and Israel and reported that higher perceived stress and fear of COVID-19 were significant predictors of PTSD, regardless of country, gender, age, and student status. Sharif-Esfahani and colleagues [11] reported that fear of COVID-19 was significantly associated with elevated levels of depression, anxiety, and PTSD among Syrian refugees. Karbasi and Eslami [12] reviewed the literature on the prevalence of PTSD among children during the pandemic and concluded that there was an increase in symptomology among children during and after the outbreak of the disease, and this was ascribed to fear of COVID-19.

Despite the prevalence of adverse mental health outcomes, many studies have provided compelling evidence of resilience and adaptation to stressors during the disease outbreak. For example, Shanahan and colleagues [13], using a longitudinal cohort study, investigated emotional distress among young Swedish adults and reported that specific coping strategies (e.g., positive reappraisal of stressors and maintaining a daily routine) were associated with lower levels of distress. A portion of young adults in their study also reported improvements in their well-being during the pandemic, and this was attributed to being removed from work and reduced educational pressures in addition to increased time spent with loved ones [13]. A critical review [14] on the impact of COVID-19 on resilience and mental health concluded that a substantial portion of the population has either remained largely unaffected or has even thrived during the pandemic. This review also reported that specific population groups, including healthcare workers, the elderly, students, and children, were particularly susceptible to stress-related psychological conditions. Kumar and colleagues [15] found that gratitude served as a significant resilience resource among college students in the United States. Students who reported higher levels of gratitude pre-pandemic were less likely to experience significant increases in anxiety during the pandemic. A study [16] assessing pre-pandemic resilience to trauma and adverse mental health outcomes during the COVID-19 pandemic among a larger sample of trauma-exposed women concluded that resilience was significantly associated with lower distress and greater emotional well-being during the pandemic. The current study focuses on a sense of coherence as a potential protective resource in the associations among fear of COVID-19, perceived stress, and the dimensions of PTSD.

Sense of coherence (SOC) [17] is a foundational concept within the salutogenic theory, introduced by Antonovsky [18] in the late 20th century. The salutogenic model is anchored in the exploration of the conditions and factors that support human health and well-being, rather than those that facilitate the onset of disease. It is predicated on the belief that health is not merely the absence of illness but a positive state of physical, mental, and social well-being. The salutogenic approach encourages a dynamic understanding of health, acknowledging that individuals continuously navigate along a health-ease versus dis-ease continuum [18]. SOC is conceptualized as a trait and encapsulates a person’s enduring confidence in the comprehensibility, manageability, and meaningfulness of life events and challenges. Comprehensibility refers to the belief that events in one’s life are predictable and explicable [17]. Manageability emphasizes the individual’s perception that they possess the resources—both internal and external—to cope with the demands of these events [17]. Meanwhile, meaningfulness stresses the idea that life’s challenges are worthy of engagement and investment. Collectively, a strong SOC reflects an individual’s ability to effectively navigate and interpret adversities, promoting health and well-being [17]. A SOC is believed to develop early in life as a result of formative experiences and is purported to remain relatively stable across an individual’s lifespan, but studies have noted that this trait-like construct increases with age [19].

In the context of health research, the salutogenic approach, with SOC at its core, shifts the focus from factors leading to disease to those promoting health and resilience [19]. A compelling base of evidence has been established through studies undertaken prior to the pandemic on the role of a SOC in mental health outcomes among various population groups. For example, a systematic review [20] of adolescents’ SOC and health concluded that SOC was related to health in terms of the adolescents’ quality of life, health behavior, mental health, and family relationships. In a meta-analytic study, del-Pino-Casado and colleagues [21] investigated the impact of caregivers’ subjective burden on mental health and concluded that higher levels of a SOC had a significant negative effect on symptoms of anxiety and depression. SOC was found to mediate psychological distress among this population group. A cross-sectional study of Chinese university students [22] reported that SOC was negatively associated with levels of perceived stress and positively associated with academic performance. Winger and colleagues [23], in a meta-analysis study focusing on distress among cancer patients, concluded that those who viewed their lives as comprehensible, meaningful, and manageable experienced less distress.

Prior research on SOC has predominantly focused on acute stressors (e.g., cancer or the subjective burden of care) that are universally experienced across various societies. However, the unprecedented nature of the COVID-19 pandemic introduced a distinct and widespread chronic stressor, transcending specific populations and geographical boundaries. The pervasive nature of the COVID-19 disease outbreak, which affected individuals from diverse backgrounds and communities, represents an opportunity to understand the role and influence of a SOC in the face of a ubiquitous threat. Given that a SOC offers insights into how individuals perceive, manage, and find meaning in life’s challenges [17], it is crucial to explore its role during global experiences of adversities to potentially inform future interventions and health strategies. The current study examines the interrelationship between the fear of COVID-19, perceived stress, a SOC, and the dimensions of PTSD.

We reasoned that higher levels of fear of COVID-19 could potentially lead to higher levels of perceived stress and that SOC would mediate the impact of fear of COVID-19 and perceived stress on the dimensions of PTSD. In this regard, we hypothesized that:

**H_1_:**  *SOC has a direct effect on the dimensions of PTSD.*

**H_2_:**  *SOC mediates the relationship between fear of COVID-19 and the dimensions of PTSD.*

**H_3_:**  *SOC mediates the relationship between perceived stress and the dimensions of PTSD.*

**H_4_:**  *The relationship between fear of COVID-19 and the dimensions of PTSD is mediated by serial perceived stress and SOC.*

## 2. Materials and Methods

### 2.1. Participants and Procedure

Participants were students (n = 322) at a university in Cape Town, South Africa. Google Forms were used to develop an online survey instrument. The electronic link was sent to a random sample of 1700 students via email, as G*Power indicated that 262 participants were required (effect size 0.2, *α* = 0.05, 1 − *β* = 0.95). The email also contained a description of the aims of the study as well as an invitation to participate. The majority of the sample were women (77%) and most of them resided in urban areas. The mean age of the sample was 26.01 years (*SD* = 10.19). Data collection occurred in the period March–July 2022 and therefore also included COVID-19-related information. In this regard, 80.1% of the sample indicated that they knew people who had contracted COVID-19, 86.6% were vaccinated, and 40.7% had lost a family member due to COVID-19.

### 2.2. Measures

Participants completed a brief demographic questionnaire as well as the following instruments: the Fear of COVID-19 Scale (FCV-19S) [24], the Perceived Stress Scale (PSS) [25], the 13-item Sense of Coherence Scale (SOC-13) [18], and the Posttraumatic Stress Disorder Checklist for DSM-5 (PCL-5) [26].

The FCV-19S is a seven-item scale that assesses emotional fear reactions related to COVID-19. Responses to the items were made on a five-point scale that ranges from “strongly disagree” (1) to “strongly agree” (5). An example of an item from the FCV-19S is “My hands become clammy when I think about coronavirus-19”. The authors of the scale reported satisfactory internal consistency (*α* = 0.82) and provided evidence of concurrent validity [24]. A South African study used a combination of classical test theory and item response theory and demonstrated that the FCV-19S is a unidimensional measure of fear of COVID-19 with satisfactory reliability (*α* = 0.91, Mokken scale reliability = 0.92) and provided evidence for construct, convergent, and concurrent validity [27]. For the current study, the FCV-19S demonstrated satisfactory reliability (*α* = 0.87).

The PSS is a 10-item measure of the extent to which respondents appraise events in their lives as stressful. The items are scored on a five-point scale, with scale anchors of “never” (0) and “very often” (4). An example of an item on the PSS is “How often have you felt difficulties were piling up so high that you could not overcome them?” The authors of the PSS reported a satisfactory estimate of internal consistency (*α* = 0.78) and provided evidence for construct validity [25]. The scale was previously used in South Africa with university students, and one study reported a Cronbach’s alpha of 0.87 for the scale [28]. The PSS had sound reliability in the current study (*α* = 0.85).

The SOC-13 is a measure of the extent to which respondents view situations in life as meaningful, manageable, and comprehensible. Responses to the 13 items are made on a seven-point scale with different scale anchors for the 13 items. An example of an item of the SOC-13 is “Do you have the feeling that you are in an unfamiliar situation and don’t know what to do?” with scale anchors of “very often” (1) and “very seldom or never” (7). The author of the scale provided a review of the psychometric properties of the scale and found reliability coefficients ranging between 0.74 and 0.91, and evidence for construct and criterion validity across 16 studies [18]. In South Africa, the SOC-13 was used with both a student [29] and teacher sample [30], yielding reliability coefficients of 0.814 in both samples. The reliability of the SOC-13 was satisfactory in the current study (*α* = 0.83).

The PCL-5 is a 20-item measure of the 20 symptoms of PTSD listed in the Diagnostic and Statistical Manual of Mental Disorders (Fifth Edition) [31]. The 20 symptoms are grouped into four symptom clusters: re-experiencing (five items), which includes recurrent and intrusive memories of, as well as nightmares and flashbacks about, the traumatic event; avoidance (two items), which includes avoidance of memories, thoughts, feelings, or external reminders about the traumatic event; negative alterations in cognitions and mood: inability to remember key features of the traumatic event and persistent and exaggerated negative beliefs or expectations about oneself or the world; hyper-arousal: irritable behavior and angry outbursts, typically expressed as verbal or physical aggression toward people or objects; hypervigilance; and exaggerated startle response [31]. In the initial validation study, the authors provided satisfactory estimates of internal consistency (*α* = 0.94) and convergence (*r*s = 0.74 to 0.85) as well as discriminant (*r*s = 0.31 to 0.60) validity [26]. A South African study reported a reliability coefficient of 0.93 for the PCL-5 in a sample of students [32]. In the current study, the subscales of the PCL had satisfactory internal consistency: re-experiencing (*α* = 0.89), avoidance (*α* = 0.89), negative alternations in mood (*α* = 0.88), and hyper-arousal (*α* = 0.82).

### 2.3. Ethical Considerations

The Humanities and Social Sciences Ethics Committee of the University of the Western Cape (ethics reference number: HS22/2/9, February 2022) approved the study. The study was conducted in line with the guidelines of the Declaration of Helsinki. Participants provided informed consent on the landing page of the electronic survey. Participation was voluntary, and no identifying particulars were collected.

### 2.4. Data Analysis

IBM SPSS for Windows Version 28 (IBM Corp., Armonk, NY, USA) was used to obtain descriptive statistics (means and standard deviations), indices of kurtosis and skewness, intercorrelations between study variables (Pearson’s r), and reliabilities (alpha) of all the instruments. With respect to skewness and kurtosis, it is suggested that skewness values between −2 and +2, as well as kurtosis values between −7 and +7, indicate that the data are approximately normally distributed [33]. Gender differences with respect to the outcome variable were examined using a two-sample *t*-test, and the relationship between age and the outcome variable was examined using Pearson’s r. Due to the relatively large sample size, we evaluated the correlation coefficients in terms of effect size and, in this regard, used the guidelines provided by Cohen [34]: small = 0.10, medium = 0.30, and large = 0.50.

We used IBM SPSS Amos for Windows Version 28 (IBM Corp., Armonk, NY, USA) to conduct a path analysis with the dimensions of PTSD as the outcome variables and perceived stress and SOC as the mediators. The significance of the direct and indirect effects was evaluated using 95% bootstrapped confidence intervals and *p*-values.

## 3. Results

The descriptive statistics (means and standard deviations), indices of skewness and kurtosis, intercorrelations between study variables (Pearson’s r), and reliabilities of scales (Cronbach’s alpha) are reported in Table 1.

The indices of skewness (−0.18 to 0.35) and kurtosis (−1.18 to −0.16) were within an acceptable range and confirmed that the data were approximately normally distributed. All the scales demonstrated satisfactory reliability (*α* = 0.82 to 0.89). Fear of COVID-19 was significantly positively associated with re-experiencing (*r* = 0.28, *p* < 0.001, small effect), avoidance (*r* = 0.26, *p* < 0.001, small effect), negative alterations in mood and cognition (*r* = 0.26, *p* < 0.001, small effect), and hyper-arousal (*r* = 0.28, *p* < 0.001, small effect). Thus, higher levels of fear of COVID-19 were associated with higher levels of PTSD. Similarly, perceived stress was significantly positively associated with re-experiencing (*r* = 0.54, *p* < 0.001, large effect), avoidance (*r* = 0.45, *p* < 0.001, medium effect), negative alterations in mood and cognition (*r* = 0.65, *p* < 0.001, large effect), and hyper-arousal (*r* = 0.63, *p* < 0.001, large effect). Higher levels of perceived stress were thus associated with higher levels of PTSD. SOC was significantly negatively associated with re-experiencing (*r* = −0.51, *p* < 0.001, large effect), avoidance (*r* = −0.45, *p* < 0.001, medium effect), negative alterations in mood and cognition (*r* = −0.69, *p* < 0.001, large effect), and hyper-arousal (*r* = −0.63, *p* < 0.001, large effect). Higher levels of a SOC were therefore associated with lower levels of PTSD.

There were no significant gender differences with regard to re-experiencing (*t* = 1.79, *p* = 0.08), avoidance (*t* = 1.45, *p* = 0.15), negative alterations in mood and cognition (*t* = 0.82, *p* = 0.41), and hyper-arousal (*t* = 1.08, *p* = 0.28). Age was significantly negatively correlated with re-experiencing (*r* = −0.21, *p* < 0.001, small effect), avoidance (*r* = −0.20 *p* < 0.001, small effect), negative alterations in mood and cognition (*r* = −0.29, *p* < 0.001, small effect), and hyper-arousal (*r* = −0.26, *p* < 0.001, small effect).

The results of the path analysis of the serial mediation model are reported in Table 2. Given the significant relationships between age and the dimensions of PTSD, age was added as a covariate in the model.

Table 2 confirms the results of the zero-order correlations between SOC and the dimensions of PTSD. In this regard, SOC had a direct effect on re-experiencing (*β* = −0.27, *p* < 0.001), avoidance (*β* = −0.27, *p* < 0.001), negative alterations in mood and cognition (*β* = −0.45, *p* < 0.001), and hyper-arousal (*β* = −0.37, *p* < 0.001). These results regarding the direct effects of a SOC of the dimensions of PTSD confirm Hypothesis 1.

Table 2 further shows that SOC did not mediate the relationship between fear of COVID-19 and re-experiencing (*β* = 0.01, *p* = 0.373), avoidance (*β* = 0.01, *p* = 0.383), negative alterations in mood and cognition (*β* = 0.02, *p* = 0.411) and hyper-arousal (*β* = 0.01, *p* = 0.408). Thus, Hypothesis 2 was rejected.

Table 2 also indicates that a SOC mediates the relationship between perceived stress and re-experiencing (*β* = 0.17, *p* = 0.001), avoidance (*β* = 0.17, *p* = 0.001), negative alterations in mood and cognition (*β* = 0.29, *p* < 0.001), and hyper-arousal (*β* = 0.24, *p* = 0.001). These results confirm Hypothesis 3.

Finally, Table 2 shows that serial perceived stress and a SOC mediates the relationship between fear of COVID-19 and re-experiencing (*β* = 0.04, *p* < 0.001), avoidance (*β* = 0.04, *p* < 0.001), negative alterations in mood and cognition (*β* = 0.06, *p* < 0.001), and hyper-arousal (*β* = 0.05, *p* < 0.001). These results confirm Hypothesis 4.

**Figure 1:**
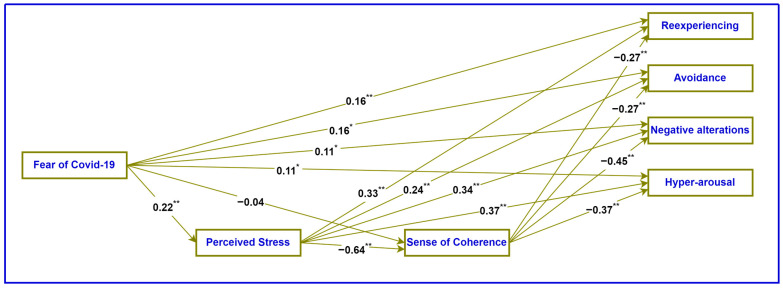
Path analysis model of the mediating role of serial perceived stress and sense of coherence. * *p* < 0.01, ** *p* < 0.001.

The path analysis model with standardized regression coefficients is presented in Figure 1.

## 4. Discussion

This study aimed to examine the association between fear of COVID-19, perceived stress, SOC, and the dimensions of PTSD. Rooted in the premise that a heightened fear of COVID-19 may invariably precipitate increased perceived stress, we sought to determine the mediating role of a SOC in this association. There were several salient findings.

First, higher levels of perceived stress and fear of COVID-19 were positively associated with PTSD symptom clusters and overall PTSD. This suggests that individuals with greater perceived stress and fear of COVID-19 were more likely to relive traumatic events related to the pandemic and avoid reminders of the experience. This could translate to avoiding discussions about the pandemic, staying away from news reports, or avoiding places or activities that may have been triggers [35]. They were also likely to experience a sense of detachment or alienation, which can disrupt interpersonal relationships, as individuals may feel estranged from their loved ones or society at large. Persistent negative emotions, such as sadness, anger, or guilt, can also pervade their daily lives [10,35]. This finding is supported by the existing literature. For example, Ikizer and colleagues [36] reported that heightened levels of perceived stress predicted greater PTSD symptomology in a sample of Turkish adults. Ochnik and colleagues [10] reported that perceived stress and fear of COVID-19, as well as fear of vaccination and trust in institutions, were significant positive predictors of COVID-19-related PTSD. These authors suggested that the uncertainty and novelty of the pandemic contributed to elevated levels of fear, which precipitated PTSD symptomology. Similar results were reported by Zhang and colleagues [37], who investigated the relationship between social support and PTSD among nursing students and found that greater fear of COVID-19 was associated with heightened symptoms of post-traumatic stress. The current study did not investigate the nature of the COVID-19-related traumatic event that participants were exposed to. The existing literature suggests that events that could be experienced as traumatic include the death of a loved one due to contracting the virus, infection with COVID-19, and, for healthcare workers, witnessing the high mortality rates associated with the pandemic and the high risk of exposure in their work environment [10].

Second, higher levels of a SOC were associated with lower levels of PTSD. This finding offers further support for existing research on the role of a SOC after trauma exposure. For example, in a meta-analytic study [38], Schäfer and colleagues found that high levels of a SOC were associated with less severe PTSD symptomology following exposure to traumatic events. Similar results have been documented in studies conducted during the pandemic. Danioni and colleagues [39], in an Italian study, found that a SOC played a protective role in the development of PTSD, irrespective of age and gender. Individuals with stronger levels of a SOC were found to be less likely to report symptoms of emotional detachment, intrusive re-experiencing, and cognitive and behavioral avoidance [39]. These researchers conjectured that individuals with a strong SOC were more able to activate perceived social support from family and friends when coping with trauma, thereby impacting the development of PTSD. A cross-sectional investigation of German COVID-19 survivors [40] revealed that among those with minimal traumatic stress symptoms, their SOC consistently remained high, even after being diagnosed with COVID-19. This interaction remained significant, even after controlling for education, age, and gender [40].

Dominant cognitive behavioral models of PTSD [41,42] ascribe the development of the disorder to faulty cognitive processing of traumatic events in memory, combined with maladaptive coping strategies and dysfunctional cognitive appraisals. A strong SOC, rooted in the ability to perceive life events as comprehensible, manageable, and meaningful, can thus serve as a protective factor. It may allow individuals to process traumatic experiences in a more adaptive manner, integrating these events into their existing worldview without succumbing to debilitating distress [41]. By fostering an understanding of the traumatic event, providing a belief in one’s capacity to manage its implications, and finding a sense of purpose or meaning amidst the adversity, a strong SOC can counteract some of the negative cognitive and emotional trajectories that often culminate in PTSD. It is also probable that those with a higher SOC are better able to mobilize internal and external resources (e.g., social support) to process the traumatic event, thereby reducing its impact [38]. In essence, the findings suggest that a SOC can potentially act as a buffer, mitigating the impact of traumatic experiences and possibly offering a foundation for recovery. 

Third, the results suggest a notable relationship between age and PTSD symptom clusters in older participants who reported fewer symptoms. The mean age of the sample was 26 years, and the aforementioned finding can be attributed to older participants possibly having more life experiences and coping mechanisms that enabled them to manage and process traumatic events more effectively than their younger counterparts. This accrued resilience could be a product of having faced and navigated past adversities, equipping them with better emotional and cognitive tools to deal with the challenges posed by the pandemic [43,44]. Furthermore, older university students might have had an advantage when it came to navigating the abrupt shifts in educational dynamics brought about by the pandemic. With more years of academic exposure, these students may have been better equipped in terms of time management, self-regulation, and study strategies, enabling them to adapt to the changes in learning modalities, such as transitioning to remote or online instruction [29]. Additionally, their previous experiences in handling academic pressures and life’s uncertainties might have granted them a broader perspective, aiding them in contextualizing the challenges of the pandemic [29,45]. 

Fourth, the results of the path analysis found that SOC had a direct effect on PTSD symptom clusters and mediated the relationship between perceived stress and symptoms of PTSD. Furthermore, serial perceived stress and SOC mediated the relationship between fear of COVID-19 and dimensions of PTSD. This means that fear of COVID-19 can increase perceived stress, which in turn, based on the individual’s SOC, can influence the development of PTSD symptoms. These findings suggest that individuals with a stronger SOC are less likely to develop symptoms associated with PTSD, despite being exposed to potential trauma, which further supports the findings in the extant literature [46,47]. The mediation effect can be attributed to the role of a SOC in helping individuals interpret and manage stressful situations more effectively. A higher SOC can act as a buffer, decreasing the impact of perceived stress on one’s mental well-being and thereby reducing the likelihood of PTSD symptoms manifesting. This finding provides an understanding of the potential psychological pathways through which pandemic-related fears can manifest into more severe mental health outcomes, such as PTSD symptoms.

Contrary to our expectations, a SOC did not significantly mediate the relationship between fear of COVID-19 and various dimensions of PTSD. This outcome suggests that the presence of a robust SOC, though pivotal in providing resilience against stress and trauma, may not directly influence the specific PTSD pathways triggered by the fear of COVID-19. The absence of mediation might be due to the unique and unprecedented nature of the COVID-19 pandemic, which introduced high levels of uncertainty, disrupted normalcy, and presented existential threats that individuals across the globe grappled with. The specific fears surrounding COVID-19, such as the fear of contracting the virus or spreading it to loved ones, may evoke a distinct stress response, leading to PTSD symptoms irrespective of the level of a SOC. 

These findings have potential implications. This study provides further empirical evidence suggesting that a stronger SOC can mediate the impact of perceived stress and pandemic-related fear, thus providing an argument for the development of resilience-focused interventions and preventive strategies. The observation of age-related resilience can contribute to developmental theories on stress and trauma, suggesting that accrued life experiences and developed coping mechanisms in older individuals can serve as protective factors against PTSD symptomatology. Given the significant association between perceived stress, SOC, and PTSD symptom clusters, there is a clinical imperative to develop interventions aimed at bolstering a SOC and managing stress more effectively. This can involve cognitive behavioral strategies, psychoeducation, and resilience-building activities. Tailored interventions can be developed for different age groups, focusing on enhancing coping mechanisms and stress management strategies in younger populations, given their observed vulnerability to developing PTSD symptoms in the context of the pandemic.

This study had certain limitations. The study utilized a cross-sectional design, which captures data at a single point in time. This limited the ability to infer causality or track changes in perceptions, stress, SOC, and PTSD symptoms over time. A longitudinal approach might have provided additional insights into the evolution of these aspects over the course of the pandemic. Data collection in this study relied on self-reported measures, which can be susceptible to response bias, recall bias, or social desirability bias. The sample consisted primarily of university students with a mean age of 26 years. This may not be representative of the general population, limiting the generalizability of the findings to broader demographic groups, including older adults, non-students, or individuals from different socioeconomic backgrounds. Other unmeasured variables, such as pre-existing mental health conditions, coping strategies, social support levels, or exposure to previous traumas, might have influenced the observed relationships and were not accounted for in this study. While path analysis offers insights into potential causal relationships between variables, it is based on correlational data. Thus, it does not establish definitive causality. Furthermore, this study was undertaken during a specific phase of the pandemic. As public perceptions, government policies, and the overall pandemic situation have since evolved, the findings may vary if the study was replicated at a different point in time.

## 5. Conclusions

In the context of the unprecedented challenges posed by the COVID-19 pandemic, this study sought to examine the relationships between fear of COVID-19, perceived stress, a SOC, and PTSD symptoms. Our findings underline the substantial role of perceived stress and fear of COVID-19 in exacerbating PTSD symptom clusters. In our study, a strong SOC emerged as a potent buffer against the development of PTSD. The inverse relationship between age and PTSD symptoms suggested that accumulated life experiences and coping mechanisms might confer a degree of resilience against traumatic events like the pandemic. The path analysis underscores the significance of a SOC, not only as a standalone protective factor but also as a mediator in the interplay between perceived stress, fear of COVID-19, and PTSD symptoms.

## Figures and Tables

**Table 1:** Intercorrelations, descriptive statistics, and reliabilities.

Variables and Indices	1	2	3	4	5	6	7
1. Fear of COVID-19	—						
2. Perceived stress	0.22 **	—					
3. Sense of coherence	−0.18 *	−0.65 **	—				
4. Re-experiencing	0.28 **	0.54 **	−0.51 **	—			
5. Avoidance	0.26 **	0.45 **	−0.45 **	0.66 **	—		
6. Negative alterations	0.26 **	0.65 **	−0.69 **	0.72 **	0.59 **	—	
7. Hyper-arousal	0.26	0.63	−0.63	0.66	0.54 **	0.80	—
Mean	17.4	23.9	47.6	9.5	4.3	13.5	11.2
SD	6.5	6.3	12.9	5.5	2.6	7.5	6.0
Skewness	0.35	−0.18	0.23	0.04	−0.21	0.02	0.02
Kurtosis	−0.44	−0.18	−0.16	−0.95	−1.18	−1.04	−0.91
Alpha	0.87	0.85	0.83	0.89	0.89	0.88	0.82

* *p* < 0.01 ** *p* < 0.001.

**Table 2:** The direct and mediating role of sense of coherence.

Effect	Beta	SE	95% CI	β	*p*
Direct effects					
Sense of coherence → Re-experiencing	−0.11	0.03	[−0.16, −0.06]	−0.27	<0.001
Sense of coherence → Avoidance	−0.05	0.01	[−0.07, −0.03]	−0.27	<0.001
Sense of coherence → Negative alterations	−0.26	0.03	[−0.31, −0.21]	−0.45	<0.001
Sense of coherence → Hyper-arousal	−0.17	0.02	[−0.21, −0.13]	−0.37	<0.001
Indirect effects					
Perceived stress → Sense of coherence → Re-experiencing	0.14	0.04	[0.08, 0.22]	0.17	0.001
Perceived stress → Sense of coherence → Avoidance	0.07	0.02	[0.04, 0.10]	0.17	0.001
Perceived stress → Sense of coherence → Negative alterations	0.34	0.05	[0.26, 0.43]	0.29	<0.001
Perceived stress → Sense of coherence → Hyper-arousal	0.22	0.04	[0.16, 0.29]	0.24	0.001
Fear of COVID-19 → Sense of coherence → Re-experiencing	0.01	0.01	[−0.01, 0.03]	0.01	0.373
Fear of COVID-19 → Sense of coherence → Avoidance	0.00	0.01	[−0.00, 0.01]	0.01	0.383
Fear of COVID-19→ Sense of coherence → Negative alterations	0.02	0.02	[−0.02, 0.06]	0.02	0.411
Fear of COVID-19 → Sense of coherence → Hyper-arousal	0.01	0.02	[−0.01, 0.04]	0.01	0.408
Fear of COVID-19 → Perceived stress → Sense of coherence → Re-experiencing	0.03	0.01	[0.02, 0.05]	0.04	<0.001
Fear of COVID-19 → Perceived stress → Sense of coherence → Avoidance	0.02	0.01	[0.01, 0.03]	0.04	<0.001
Fear of COVID-19 → Perceived stress → Sense of coherence → Negative alterations	0.07	0.02	[0.04, 0.11]	0.06	<0.001
Fear of COVID-19 → Perceived stress → Sense of coherence → Hyper-arousal	0.05	0.01	[0.03, 0.07]	0.05	<0.001

## Data Availability

The data that support the findings of this study are available from the corresponding author, upon reasonable request.
